# Knowledge and awareness about human papillomavirus infection and its vaccination among women in Arab communities

**DOI:** 10.1038/s41598-020-80834-9

**Published:** 2021-01-12

**Authors:** Mervat M. Alsous, Ahlam A. Ali, Sayer I. Al-Azzam, Mariam H. Abdel Jalil, Hala J. Al-Obaidi, Esraa I. Al-abbadi, Zainab K. Hussain, Feras J. Jirjees

**Affiliations:** 1 Department of Clinical Pharmacy and Pharmacy Practice, Faculty of Pharmacy, Yarmouk University, Irbid, Jordan; 2 Medical Biology Centre, School of Medicine, Dentistry and Biomedical Sciences, Queen’s University Belfast, Belfast, UK; 3 Department of Clinical Pharmacy, Faculty of Pharmacy, Jordan University of Science and Technology, Irbid, Jordan; 4 Department of Biopharmaceutics and Clinical Pharmacy, Faculty of Pharmacy, University of Jordan, Amman, Jordan; 5 Clinical and Practice Research Group, School of Pharmacy, Queen’s University Belfast, Belfast, UK; 6 Qatar National Cancer Registry, National Cancer Program, Ministry of Public Health, Doha, Qatar; 7 Department of Biology, College of Science, University of Baghdad, Baghdad, Iraq; 8 Department of Pharmacy Practice and Pharmacotherapeutics, College of Pharmacy, University of Sharjah, Sharjah, United Arab Emirates

**Keywords:** Cancer, Health care

## Abstract

Cervical cancer (CC) is one of the most common types of cancer that affect females worldwide with hundreds of thousands of women dying annually due to this disease, mainly in developing countries. Infection with human papillomavirus (HPV) is the main risk factor for this cancer. There are no public awareness and national immunization programs in most Arab countries. This study aimed to investigate the knowledge and awareness about the HPV vaccine among females in four Arab countries and their acceptance to receive the vaccine. A cross-sectional study was conducted in several Arab countries: Jordan, Qatar, the United Arab Emirates (UAE), and Iraq. Respondents that fulfilled the desired criteria and were willing to participate in the study were asked to fill out the survey. Knowledge and awareness were assessed using 13 questions. Ethical approvals were given from the four countries. A total of 3658 individuals participated in the study; however, 2804 responses were included in the analysis and more than one third of participants (n = 1007) were aged between 18 and 25 years old. This study revealed poor awareness and knowledge of the participants about HPV and its vaccine among all four countries’ participants with relatively better knowledge among participants from the UAE. Participants who are younger (18–25 years old), have a postgraduate education, have an education or career related to the medical field, or had a Pap smear in the last three years tend to have higher knowledge about the HPV vaccine compared to others. Poor knowledge and awareness findings in this study were expected, considering the lack of public education campaigns regarding the HPV virus coupled with the absence of the HPV vaccination from the national immunization schedule in three participating countries (Jordan, Qatar, and Iraq). It is recommended that there is a need to provide national educational campaigns about the HPV vaccine to the public in all Arab populations.

## Introduction

Cervical cancer (CC) is one of the greatest threats to women’s health as one woman dies every two minutes due to this disease^1^. It is the fourth most common type of cancer in females worldwide and every year several hundred thousand women die because of this disease, mainly in developing countries^2,3^.

One of the main risk factors for CC worldwide is infection with human papillomavirus (HPV), which is the most common sexually transmitted infection. This virus might also cause other anogenital cancers and many health problems in both genders^2^.

In 2006, the first vaccine against HPV was approved by the Food and Drug Administration (FDA) of the United States of America for the primary prevention of CC. Then, two more prophylactic vaccines against HPV were also registered. The HPV vaccination is routinely recommended for adolescents at age 11 or 12 years old and can be given as early as 9^4^. The World Health Organization (WHO) recommends HPV vaccines as part of routine vaccinations in all countries^5^. Currently, these vaccines are highly used worldwide, and it is considered part of the national vaccination programs in 105 countries^6^.

Globally, there are several factors which might influence the slow uptake of HPV vaccines, including financial constraints, weak infrastructure for the adolescent vaccine delivery, lack of reliable data on the burden of the HPV disease, and cultural and religious sensitivities related to this topic^7^.

In Arab countries, more than ten thousand women were diagnosed with CC (with 7365 deaths) in 2018^8^. These numbers are expected to increase dramatically in a few decades unless effective public health interventions are introduced^9^. In addition, studies conducted in Arab societies indicated that the prevalence rate of HPV was 16% in the general population and 80% in CC patients^2,10,11^. Moreover, there is no HPV vaccination program implemented in these countries, except in the United Arab Emirates (UAE) which has a preliminary HPV program^12^. In addition, there is no official CC screening program and the information related to this health issue is mainly collected from opportunistic screening tests in Arab societies^13–15^. Generally, the societies in the Arab countries are characterized by sharing relatively comparable cultures and religious conservatism^7^. This implies a conservative sexual behavior. Moreover, discussing these issues is relatively very low among people in the society with no formal sexual education including health sexual education in most Arab countries^16^. Therefore, the people in Arab countries are facing a threatening health problem of increased CC. This is mainly due to the lack of effective CC screening programs and vaccination programs as well. In addition, there is low knowledge and attitude among people relating to the HPV vaccine in some Arab societies^7,17–19^.

The aim of the study was to evaluate the knowledge about the HPV infection, the awareness towards the HPV vaccination, and the perceived barriers among women in four Arab countries: Jordan, Iraq, the UAE, and Qatar. Furthermore, the aim was to assess public acceptance and willingness to receive the vaccine.

## Methodology

### Study design

The present study is a cross-sectional survey, which was intended to investigate the knowledge and awareness about HPV infection, its correlation to cervical cancer, and its vaccine among females in the Arab region.

### Ethics and subject recruitment

The study was conducted after obtaining ethical approval from the Institutional Review Board (IRB) of Jordan University of Science and Technology (JUST) and King Abdulla University Hospital (KAUH) in Irbid, Jordan (201/132/2020). The protocol of the study was approved by the Health Research Governance Department at the Ministry of Public Health (MoPH) as Exempt Research in Qatar. Moreover, the study was approved by the Research Ethical Committee at the University of Sharjah in the United Arab Emirates (REC-20–05-17–01) and the Scientific Research Committee at the University of Baghdad in Iraq (3311/4–2020/6/29). All methods were carried out in accordance with relevant guidelines and regulations.

The study was conducted in the four Arab countries of Jordan, Qatar, the United Arab Emirates (UAE) and Iraq. Females aged ≥ 18 years from these countries were invited to participate in the study. Exclusion criteria were (a) males, (b) participants living in countries other than those included in the study, and (c) respondents less than 18 years old.

The questionnaire was distributed utilizing the electronic format Google Forms. The link to the survey which was preceded by an introductory section about the study was sent via various social media platforms. The introductory section described the aim and objectives of the study and the voluntary nature of participation with a consent statement if they would like to take part in the study. Therefore, consent to participate was considered taken if the respondent signed the consent form electronically and filled out the questionnaire. Informed consent was obtained from all study participants. The questionnaire was terminated automatically if participants declined to take part or if they were males or younger than 18 years old. The participants were requested to complete the survey without consulting people, materials, textbooks, or internet web pages. All methods were approved by the IRB committee.

### Study instruments

The questionnaire utilized to collect data from the females was developed by the authors via an extensive review of the literature. The final version of the questionnaire was composed of three sections. The first section was designed to collect general demographic data about the participants such as age, gender, occupation, and level of education. The second section included 13 questions aimed to evaluate the participants’ general knowledge and awareness about HPV and its vaccine. These questions were related to the mode of transmission of virus, its correlation to CC, and the vaccine benefit, availability, target population, and side effects. Each answer scored as correct or incorrect. The respondent was given a zero for each wrong answer and one point for each correct answer, and the results were summed to give a total score out of 13. Participants who scored > 50% were considered to have good knowledge and who scored 50% and less considered as having a poor level of knowledge/awareness.

The third section intended to assess the public acceptance to receive the HPV vaccine and willingness to recommend it to a child or adolescent (age between 9 and 12 years old) or friend. Another part of this section was related to concerns about the vaccine, including worries regarding side effects, efficacy, and the financial burden. The questionnaire was in the Arabic language and reviewed by the authors and then subjected to a pilot testing by 40 participants to ensure the clarity of the questions, which resulted in several minor amendments.

### Statistical analysis

#### Sample size calculation

The target sample size was estimated based on the Raosoft software sample size calculator for the minimal sample size needed for an unlimited population size using a confidence interval of 95%, a standard deviation of 0.5, and a margin of error of 5%. The required sample size was 385 participants from each study population.

#### Statistical analysis

Data were analyzed using the Statistical Package for the Social Sciences (SPSS) version 22. Respondents’ demographic characteristics were described using descriptive statistics. Differences between categorical data were detected using a chi-square test. Continuous data was reported as median (interquartile range; IQR) for non-normally distributed variables. The relationship between the knowledge score and the independent categorical variables was determined using the Mann–Whitney U test for binomial variables and the Kruskal–Wallis test for multinomial variables. Variables that were significantly associated with the knowledge score were included in the multiple linear regression analysis after log transformation of the knowledge score. A statistical significant difference was considered if the p-value was less than 0.05.

#### Excluding careless responses

To exclude careless answers, we added a question at the end of the questionnaire if participants would recommend including their response in the analysis. This approach was utilized, as suggested by Maede et al., as a Self‐Reported Single Item (SRSI) Indicator^20^.

## Results

### Participants’ characteristics

A total of 3658 individuals participated in the study, of which 854 were excluded from the analysis (19 declined to take part, 235 reported careless responses, 167 were males, 49 were less than 18 years old, and 384 were from countries other than those included in the study). Thus, in the present analysis, we analyzed a total of 2804 responses of which 1216 were from Jordan, 397 were from Qatar, 606 from the UAE, and 585 from Iraq. Around one third of participants (n = 1007, 35.9%) were aged between 18 and 25 years. The largest proportion of the participating females were unemployed (n = 1363, 48.6%). About half of the participating females (n = 1419, 50.6%) were studying non-medical sciences. General demographic data of the included participants are presented in Table 1.

**Table 1:** Participants' demographic data (n = 2804).

	Overall (n = 2804)	Jordan (n = 1216)	Qatar (n = 397)	UAE (n = 606)	Iraq (n = 585)
**Age (years)**					
18–25	1007 (35.9)	369 (30.4)	137 (34.5)	229 (37.8)	273 (46.7)
26–35	843 (30.1)	399 (32.8)	150 (37.8)	159 (26.2)	135 (23.1)
36–45	660 (23.5)	303 (24.9)	76 (19.1)	164 (27.1)	116 (19.8)
46–55	229 (8.2)	113 (9.3)	26 (6.6)	41 (6.8)	49 (8.4)
≥ 56	65 (2.3)	32 (2.6)	8 (2.0)	13 (2.2)	12 (2.1)
**Occupation**					
Not employed	1363 (48.6)	546 (44.9)	198 (49.9)	398 (65.7)	221 (37.8)
Work related to Health sector	655 (23.4)	336 (27.6)	55 (13.9)	88 (14.5)	176 (30.1)
Work not related to Health field	786 (28.0)	334 (27.5)	144 (36.3)	120 (19.8)	188 (32.1)
**Educational level**					
Secondary school or less	251 (9.0)	86 (7.1)	50 (12.5)	63 (10.4)	51 (8.7)
Undergraduate study	2068 (73.8)	916 (75.3)	311 (78.3)	495 (81.7)	347 (59.3)
Postgraduate study	485 (17.3)	214 (17.6)	36 (9.1)	48 (7.9)	187 (32.0)
Educational field*					
Education related to medical field	1134 (40.4)	520 (42.8)	69 (17.4)	317 (52.3)	320 (54.7)
Education not related to medical field	1419 (50.6)	610 (50.2)	278 (70.0)	226 (37.3)	214 (36.6)
**Marital status**					
Single	1216 (43.4)	494 (40.6)	146 (36.8)	233 (38.4)	343 (58.6)
Married	1489 (53.1)	672 (55.3)	237 (59.7)	355 (58.6)	225 (38.5)
Divorced/widow	99 (3.5)	50 (4.1)	14 (3.5)	18 (3.0)	17 (2.9)
**Living place**					
Urban	2627 (93.7)	1067 (38.1)	387 (97.5)	604 (99.7)	569 (97.3)
Rural	177 (6.3)	149 (5.3)	10 (2.5)	2 (0.3)	16 (2.7)
**Family income ($)**					
< 700	591 (21.1)	335 (27.6)	34 (8.6)	69 (11.4)	154 (26.3)
700–1400	1112 (39.7)	499 (41.0)	137 (34.5)	204 (33.7)	272 (46.5)
> 1400	1100 (39.2)	382 (31.4)	226 (56.9)	333 (55.0)	159 (27.2)
**Pap smear in the last 3 years**					
Yes	527 (18.8)	208 (17.1)	76 (19.1)	200 (33.0)	43 (7.4)
Never	2158 (77.0)	972 (79.9)	296 (74.6)	365 (60.2)	525 (89.7)
Yes, more than 3 years ago	119 (4.2)	36 (3.0)	25 (6.3)	41 (6.8)	17 (2.9)
**Know someone with cervical cancer**					
Yes	243 (8.7)	98 (8.1)	29 (7.3)	52 (8.6)	64 (10.9)
No	2561 (91.3)	1118 (91.1)	368 (92.7)	554 (91.4)	554 (91.4)

*9% had educational level of secondary school or less.

### Participants’ knowledge and awareness about HPV and the vaccine

Knowledge and awareness about HPV and its vaccine were measured using 13 questions with one point for each question. The overall median knowledge score was 2 (IQR = 5) in the whole population. Table 2 displays the proportion of females who answered questions correctly related to HPV and its vaccine.

**Table 2:** Participant’s knowledge about Human Papilloma Virus and awareness about vaccine (n = 2804).

Question	Correct answers N (%)				
	Overall (n = 2804)	Jordan (n = 1216)	Qatar (n = 397)	UAE (n = 606)	Iraq (n = 585)
**Knowledge about HPV**					
1. HPV is a virus that is sexually transmitted^1^	753 (26.9)	304 (25.0)	61 (15.4)	186 (30.7)	202 (34.5)
2. HPV will usually go away on its own without treatment^1^	987 (35.2)	412 (33.9)	98 (24.7)	255 (42.1)	222 (38.0)
3. HPV causes cervical cancer^1^	1229 (43.8)	533 (43.8)	113 (28.5)	310 (51.2)	273 (46.7)
**Awareness about HPV vaccine**					
1. Have you ever heard about HPV vaccine?^1^	733 (26.1)	289 (23.8)	74 (18.6)	241 (39.8)	129 (22.1)
2. Does the HPV vaccine prevent cervical cancer?^1^	568 (20.3)	245 (20.2)	47 (11.8)	163 (26.9)	113 (19.3)
3. Does HPV vaccine protect against all types of Cervical Cancer?^2^	717 (25.6)	303 (24.9)	75 (18.9)	163 (26.9)	176 (30.1)
4. Is HPV vaccine available in your country?^1^	407 (14.5)	137 (11.3)	40 (10.1)	214 (35.3)	16 (2.7)
5. Can the HPV vaccine cause side effects?^1^	598 (21.3)	268 (22.0)	63 (15.9)	134 (22.1)	133 (22.7)
6. Can the HPV vaccine cause HPV infection?^2^	662 (23.6)	285 (23.4)	63 (15.9)	172 (28.4)	142 (24.3)
7. Does the HPV vaccine decrease the chance of having changes in the Pap smear test?^1^	568 (20.3)	262 (21.6)	47 (11.8)	136 (22.4)	123 (21.0)
8. Whom should be vaccinated with HPV vaccine? *	579 (20.7)	245 (20.2)	80 (20.2)	99 (16.3)	155 (26.5)
9. Do females need to be screened for HPV before vaccinated?^2^	255 (9.1)	99 (8.1)	30 (7.6)	76 (12.5)	50 (8.6)
10. Can it be given to a woman having HPV infection?^1^	301 (10.7)	111 (9.1)	49 (12.3)	62 (10.2)	79 (13.5)

1: Yes, 2: No, *: Males and females.

The percentages of participants who were aware that the HPV infection will not go away on its own without treatment and that the infection causes CC were 35.2% and 43.8%, respectively. Additionally, 26.9% of participants knew that the HPV infection is a sexually transmitted disease. About a quarter of participants (25.6%) were aware that the vaccine does not protect against all types of CC. Only 21.3% of participants were aware that HPV causes some side effects such as headache and nausea, and 20.3% knew that the HPV vaccine can prevent CC and decrease the chance of having changes in the Pap smear test. Moreover, the proportion of participants who were aware that the target population for vaccination is both males and females and that taking the vaccine will not infect the recipient were 20.7% and 23.6%, respectively.

The lowest median score was 1.0 (IQR = 4) for participants from Qatar while the highest median score was 3.0 (IQR = 5) for participants from the UAE.

The results of the univariate analysis are presented in the supplementary material (Supplementary Table 1), where all the 10 tested predictors were found significant. One of the significant factors (i.e., marital status: single) was removed from the multivariate analysis due to multicollinearity with the age ≤ 25 (correlation coefficient = 0.706). Therefore, only nine variables were subjected to the multivariate linear regression analysis and results showed that only six predictors were significant. Results of the multivariate regression analysis are portrayed in Table 3. The coefficient of determination (R^2^) for the current model was 0.165, which is 16.5% of the variance.

**Table 3:** Multiple linear regression analysis for variables associated with knowledge and awareness score about HPV and its vaccine (n = 2804).

Variable	Unstandardized B	SE	95% CI	P-value
Participants from UAE	0.077	0.017	0.044–0.109	< 0.001*
Participants not from UAE (reference group)				
Age ≤ 25 years	0.062	0.016	0.030–0.095	< 0.001*
Age > 25 years (reference group)				
Career related to medical field	0.077	0.017	0.043–0.111	< 0.001*
Not employed or career not related to medical field (reference group)				
Postgraduate study	0.073	0.018	0.037–0.109	< 0.001*
Undergraduate study or less (reference group)				
Education related to medical field	0.178	0.017	0.145–0.211	< 0.001*
Education not related to medical field (reference group)				
Income > 1400$	0.028	0.014	0.000– 0.055	0.050
Income ≤ 1400$ (reference group)				
Know someone with Cervical Cancer	-0.006	0.022	-0.050 -0.037	0.782
Do not Know someone with Cervical Cancer (reference group)				
Living Place (Urban)	0.016	0.028	-0.039–0.070	0.575
Living in rural Place (reference group)				
Had pap smear in the last 3 years	0.090	0.019	0.053–0.127	< 0.001*
Had pap smear > 3 years or never had pap smear (reference group)				

UAE: United Arab Emirates. *p-value < 0.05.

The multiple linear regression analysis identified the following groups to be more aware about HPV and its vaccine: (a) young participants ≤ 25 years old, (b) participants from the UAE, (c) participants who have a postgraduate degree, (d) participants with education related to the medical field, (e) participants with a career related to the medical field, and (f) participants who had a Pap smear in the last three years.

### Acceptance and concerns about HPV vaccine

Among all participants, only 3.0% (n = 84) reported that they had received the HPV vaccine. Moreover, 43.2% of participants reported that they are willing to receive the vaccine while 52.6% reported that they cannot decide whether they are willing to get the vaccine or not according to the information they have. Only 7.4% would recommend the HPV vaccine for a child or adolescent and 29.0% would recommend it for a friend or relative. Detailed responses are presented in Table 4.

**Table 4:** Participant’s acceptance of Human Papilloma Virus vaccine (n = 2804).

Question	N (%)				
	Overall (n = 2804)	Jordan (n = 1216)	Qatar (n = 397)	UAE (n = 606)	Iraq (n = 585)
**1. Are you willing to receive the HPV vaccine which can protect against HPV infection?**					
Yes	1211 (43.2)	511 (42.0)	141 (35.5)	291 (48.0)	268 (45.8)
No	118 (4.2)	50 (4.1)	11 (2.7)	27 (4.5)	30 (5.1)
Cannot decide according to my information	1475 (52.6)	655 (53.9)	245 (61.7)	288 (47.5)	287 (49.1)
**2. Would you recommend the HPV vaccine for a child or adolescent (age between 9–12 years old)?**					
Yes	208 (7.4)	86 (7.1)	15 (3.8)	57 (9.4)	50 (8.5)
No	727 (25.9)	256 (21.1)	117 (29.5)	188 (31.0)	166 (28.4)
Cannot decide according to my information	1869 (66.7)	874 (71.9)	265 (66.8)	361 (59.6)	369 (63.1)
**3. Would you recommend the HPV vaccine for a friend or relative?**					
Yes	812 (29.0)	317 (26.1)	79 (19.9)	214 (35.3)	202 (34.5)
No	104 (3.7)	32 (2.6)	17 (4.3)	26 (4.3)	29 (5.0)
Cannot decide according to my information	1888 (67.3)	867 (71.3)	301 (75.8)	366 (60.4)	354 (60.5)

**Figure 1:**
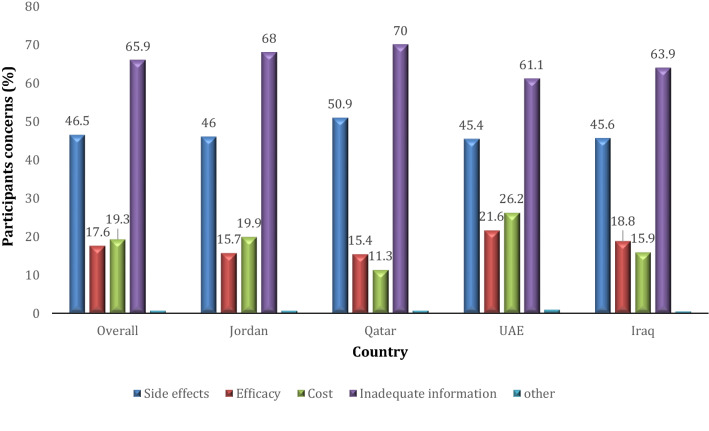
Participants concerns about receiving or recommending HPV vaccine (n = 2804).

Figure 1 illustrates the concerns about the HPV vaccination as described by participants with inadequate information (65.9%) being the main concern followed by side effects (46.5%) and the high cost of the vaccine (19.3%).

## Discussion

The current study investigated the level of knowledge and awareness about HPV and its vaccine among women living in four Arabic countries: Jordan, Qatar, the UAE, and Iraq. Collectively, the present findings suggested relatively poor awareness and knowledge of the participants about HPV and its vaccine in the four Arab communities with relatively better knowledge among participants from the UAE. This finding was expected considering the lack of public education campaigns regarding the HPV virus coupled with the absence of the HPV vaccination from the national immunization schedule in three of the participating countries (i.e., Jordan, Qatar, and Iraq).

The results regarding the awareness of the relation between the HPV infection and CC showed inadequate knowledge about CC epidemiology compared to other studies conducted on Arab and non-Arab communities^17,21,22^.

Collectively, only 26.1% of sampled participants have heard about the vaccine. This percentage is lower than what has usually been found in European countries and based on some studies even that reported in other countries with Islamic majorities such as Turkey^23,24^. Interestingly, the Ortashi et al. study in 2013 reported that 22.0% of females in the UAE have heard about the vaccine, but our results showed a much higher percentage of 38.9% for the UAE population^25^.

Oncoviruses can enable different carcinogenesis stages, with one such virus being the HPV. Although within two years of infection most HPV infections can be cleared or deactivated, high risk HPV types remain and can progress to CC^26^. The DNA of high-risk HPV was found in 99.7% of CC specimen^27^. Unfortunately, the present study results showed relatively low knowledge of this strong link between the HPV and CC among study participants, especially in Qatar. Furthermore, only about a quarter of the participants had heard about the HPV vaccine, which could explain the low knowledge of participants about its various aspects especially in its role to protect against CC. This is information that is quite important to communicate to the public, as it was reported that breast cancer, leukemia, and CC were the most common incident cancers in the Eastern Mediterranean Region^28^.

It was previously reported that being employed in the medical field was associated with higher knowledge and awareness about the HPV and its vaccine^17^. A result that was noted in the current study. We also found higher knowledge and awareness about the HPV and its vaccine among students in the medical field. Furthermore, younger age was associated with higher knowledge and awareness regarding HPV and its vaccine. A possible explanation is that the peak incidence of HPV is 25 years and that the vaccine is recommended to be taken routinely for ages 11–26 although it can be given to selected adults between 27 and 45 years old^29,30^.

Despite the relatively poor overall detected knowledge regarding the HPV and its vaccine, almost half of women who responded to this survey reported their willingness to receive the vaccine, but a smaller proportion reported recommending it to a friend or a child. Refraining from recommending the vaccine to children can be understood considering the fragility of this population coupled with the low detected level of knowledge regarding the vaccine. The willingness of women to take the HPV vaccine despite their poor knowledge about it has also been reported by other researchers in both Arab and other countries^31–34^.

In the present study, more than half of the respondents from all participating countries reported inadequate information about CC and the HPV vaccine which was the main concern about receiving or recommending the HPV vaccine. The other main concern was about the side effects which was a similar finding to a study on public from another Arab community in Bahrain^17^. Therefore, it is recommended that a national HPV awareness campaign should be stratified in Arab countries to gap the knowledge about the HPV vaccine.

To the best of our knowledge, this is the first and largest cross-sectional study (involving 2804 participants) in the Middle East that explored knowledge about the HPV infection, its correlation to CC, and awareness about the HPV vaccine. The large sample size improved the generalizability of these findings.

Limitations of the present study include the inability to calculate the response rate as the survey was distributed electronically, the possibility of selection bias due to the online nature of the survey, and the possibility of careless responses. Consequently, we may have missed some of the targeted population. However, we tried to overcome this by distributing the survey among four different populations and widely used social media. In the present study, the detected careless responses based on self-reported single item was 6.4% of the total received responses. This was higher than previous research studies (2.8%) by our group^35^. Despite this measure, the elimination of careless responses cannot be completely assured.

## Conclusion

There is limited knowledge and awareness about the HPV infection, its correlation to cervical cancer, and the HPV vaccine in Arab communities. Predominance for the willingness to take the HPV vaccine was also accompanied with concerns regarding inadequate knowledge about it.

Participants who are younger (18–25 years old), have postgraduate study, have an education or career related to the medical field, and had a Pap smear in the last three years tend to have higher knowledge and awareness about the HPV vaccine compared to others.

### Suggestions and recommendations

The results of this study reveal an insufficient level of knowledge about CC and the HPV vaccine; therefore, we recommend that there is an actual need to provide national educational campaigns about the HPV vaccine to the public among all Arab communities. We have highlighted some important gaps in information about the HPV infection and its vaccine, which can be addressed in the future.

## Supplementary Information

Supplementary Information.

## Data Availability

The data that supported the results of this study are obtainable from the corresponding author upon reasonable request.
