# Supplementary material for: Knowledge and awareness about human papillomavirus infection and its vaccination among women in Arab communities
Source: Sci Rep. 2021 Jan 12;11:786. doi: 10.1038/s41598-020-80834-9 (PMC7804285; doi:10.1038/s41598-020-80834-9)
Supplement: Supplementary file 1 — Supplementary Information. [file 41598_2020_80834_MOESM1_ESM.docx]

**Supplementary material**

**Table 1: Knowledge and awareness score stratified by participants’ characteristics (n=2804)**

|  | | **Knowledge and awareness score** | | |
| --- | --- | --- | --- | --- |
| **Variable** | **N (%)** | **Median** | **IQR** | **P-value** |
| **Country**  Jordan  Qatar  UAE  Iraq | 1216 (43.4)  397 (14.2)  606 (21.6)  585 (20.9) | 2.00  1  3.0  3.0 | 5  4  5  4 | <0.001* |
| **Age (Years)**  ≤25  >25 | 1007 (35.9)  1797 (64.1) | 3  2 | 4  5 | <0.001* |
| **Occupation**  Not employed  Career related to medical field  Career not related | 1363 (48.6)  655 (23.4)  786 (28.0) | 2  5  1 | 4  5  4 | <0.001* |
| **Educational level**  -Secondary school or less  -Undergraduate study  -Postgraduate study | 251 (9.0)  2068 (73.8)  485 (17.3) | 2  2  3 | 4  5  5.5 | <0.001* |
| **Education field**  Education related to medical field  Education not related | 1134 (40.4)  1419 (50.6) | 4  1 | 5  3 | <0.001* |
| **Living place**  Urban (highest)  Rural | 2627 (93.7)  177 (6.3) | 2  2 | 5  4 | 0.048* |
| **Marital status**  Single  Married  Widow/divorced | 1216 (43.4)  1489 (53.1)  99 (3.5) | 3  2  2 | 4  5  5 | <0.001* |
| **Income ($)**  <700  700-1400  >1400 | 591 (21.1)  1112 (39.7)  1100 (39.2) | 2  2  3 | 4  5  5 | <0.001* |
| **Having pap smear test in the last 3 years**  Yes (highest)  Never  More than 3 years ago | 527 (18.8)  2158 (77.0)  119 (4.2) | 3  2  2 | 6  5  4 | 0.004* |
| **Know someone with Cervical Cancer**  Yes  No | 243 (8.7)  2561 (91.3) | 4  2 | 5  5 | 0.002* |

*P<0.05
